# Outcomes of novel 3D-printed fully porous titanium cup and a cemented highly cross-linked polyethylene liner in complex and revision total hip arthroplasty

**DOI:** 10.1186/s42836-022-00152-5

**Published:** 2022-12-02

**Authors:** Ittai Shichman, Lyndsay Somerville, William B. Lutes, Stephen A. Jones, Richard McCalden, Ran Schwarzkopf

**Affiliations:** 1 Department of Orthopedic Surgery, NYU Langone Health, New York, NY 10010 USA; 2 Division of Orthopedic Surgery, Sourasky Medical Center, Sackler School of Medicine, Tel-Aviv University, 6423906 Tel-Aviv, Israel; 3 Adult Hip and Knee Reconstructive Surgery, London Health Sciences Centre - University Hospital, London, ON N6A 5A5 Canada; 4 Department of Orthopedic Surgery, Aurora Medical Center, Kenosha, WI 51432 USA; 5 Department of Trauma and Orthopaedics, University Hospital of Wales and University Hospital, Llandough, CF64 2XX Wales, UK; 6 Department of Orthopaedic Surgery, London Health Sciences Centre, Western University, London, ON N6A 5A5 Canada; 7 NYU Langone Health, NYU Langone Orthopedic Hospital, 301 East 17th Street, New York, NY 10003 USA

**Keywords:** Complex primary THA, Revision THA, Bone loss, Paprosky, Aseptic loosening

## Abstract

**Introduction:**

A novel fully porous acetabular titanium shell has been designed to reduce stiffness mismatch between bone and implant and promote osseointegration in complex (cTHA) and revision total hip arthroplasty (rTHA). A highly cross-linked polyethylene (XLPE) liner is cemented within the cup to reduce wear rates and increase survivorship. This study reported the outcomes of an XLPE liner cemented into a novel 3D-printed fully porous cup in cTHA and rTHA.

**Methods:**

Presented was a multicenter retrospective review of 40 patients (6 cTHA and 34 rTHA) who underwent THA with a fully porous titanium acetabular cup and cemented XLPE liner. Data were collected on demographics, surgical information, outcomes, including osseointegration and migration and implant survivorship.

**Results:**

On average, patients were 71.42 ± 9.97 years old and obese (BMI: 30.36 ± 6.88 kg/m^2^) and were followed up for a mean time of 2.21 ± 0.77 years. Six patients underwent cTHA and 34 patients underwent rTHA. The mean hospital length of stay was 5.34 ± 3.34 days. Three (7.5%) 90-day readmissions were noted. Harris Hip Scores improved, on average, from 53.87 ± 12.58 preoperatively to 83.53 ± 12.15 postoperatively (*P*<0.001). One case of acetabular shell aspetic loosening with migration was noted. Thirty-nine of the 40 acetabular components were fully osseointegrated without migration. Two patients underwent re-revision surgery for PJI and one patient received acetabular shell+liner re-revision due to aseptic loosening. Kaplan-Meier analysis showed an all-cause revision-free survival rate of 95.0% at 6 months and 1 year, and 92.0% at 4-years. Aseptic acetabular cup, liner dislocation/loosening, and fracture-free survival was 100% at 6 months and 1-year, and 97.1% at 2 years.

**Conclusion:**

The combined use of a novel 3D-printed fully porous titanium acetabular shell and cemented XLPE acetabular liner yielded excellent rates of osseointegration, and all-cause and acetabular aseptic loosening survivorship at a minimum 1-year follow-up. Further long-term studies are needed to assess the longevity of this construct.

## Introduction

Aseptic loosening (AL) with subsequent failure of the acetabular component is the most common indication for revision total hip arthroplasty (rTHA) and demand for this surgery is expected to grow substantially in the coming years [1, 2]. Major goals in the revision acetabular surgery are to achieve primary and long-term stable fixation of the shell [3]. Acetabular bone loss encountered during complex primary and revision hip arthroplasty can pose a major challenge to surgery. Cavitary bone defects are the most commonly encountered major bone defects [4]. These defects represent a volumetric loss in bony substance of the acetabular cavity including the medial wall. Several treatment options were developed through the years, including allograft and cage constructs as well as use of metal augments [3, 5, 6]. In recent years, trabecular metal revision shells became a preferred modality due to their excellent survivorship based on progressive osseointegration with the surrounding bone [3]. Traditionally, these fully porous revision shells were used with a cemented polyethylene liner, allowing the surgeon to achieve optimal positioning to reduce impingement and dislocations [3].

Lakstein *et al*. reported a similar survivorship of 96%, including improved patient-reported outcomes in a series of 53 cases of revision THA using trabecular metal acetabular shells [3]. In a more recent study, Bawale *et al*. reported a 96.0% implant survivorship in a 7.2 year follow-up of 41 revision THAs using tantalum porous metal implants [7]. Hosny *et al*. reported an acetabular shell aseptic survivorship of 98.4% at a mean follow-up of 87.6 months in 62 cases of rTHA using a titanium highly porous multihole acetabular shell [8]. Moreover, in a 108 complex primary THA cohort using a 3D-printed fully porous trabecular shell, Geng *et al*. reported a 99.1% survivorship at a 2-year minimum follow up [9].

The purpose of this study was to analyze the short-term survival and outcomes using a novel fully porous acetabular shell with a cemented XLPE liner for moderate-to-severe acetabular bones loss.

## Materials and methods

**Fig. 1:**
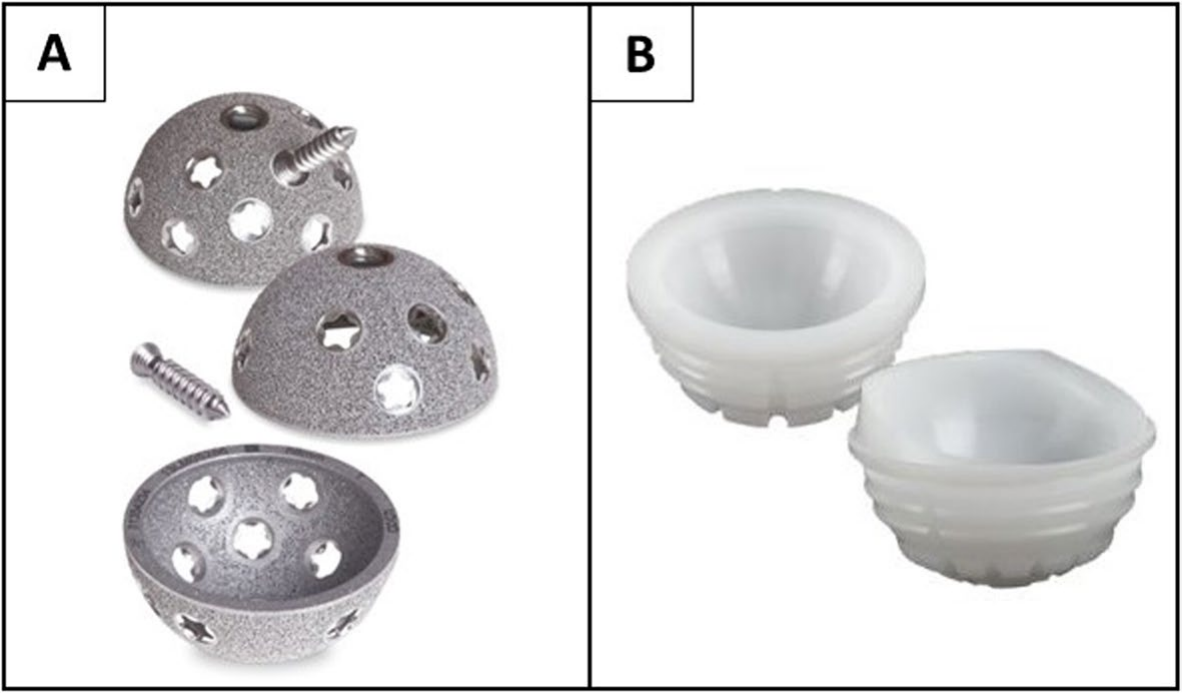
**A** Fully porous multi-hole titanium cup with variable angle locking screws. **B** Lateralized XLPE liner

After institutional review board approval, this multicenter retrospective study examined all patients who had undergone cTHA and rTHA in which the acetabular component was revised with a novel titanium fully porous shell into which a new XLPE liner was cemented (Redapt Revision Hip System, Smith&Nephew, Memphis, TN, USA) between January 2016 to November 2018 at four large institutions (NYU Langone Orthopedic Hospital, NY, USA; Department of Orthopedic Surgery, Aurora Medical Center, Kenosha, WI, USA; Department of Trauma and Orthopaedics, University Hospital of Wales and University Hospital Llandough, Wales, UK; Adult Hip and Knee Reconstructive Surgery; London Health Sciences Centre - University Hospital, London, Canada) (Fig. 1). Fully porous shells with a cemented XLPE liner were used in the cases of acetabulum bone defects in complex primary (defined as primary THA in patients with compromised acetabular bone quality) and revision THAs.

Inclusion criteria for this study included: complex primary and revision THA and patients aged 18-years or older. Exclusion criteria included oncologic lesions in the affected hip joint and patients who were not involved in a postoperative follow-up imaging study.

Forty-eight patients were initially screened. Five patients were lost to follow-up and 3 did not complete a minimum follow-up time of 1 year. In total, 40 patients completed at least 1 year of follow-up and were included in this study. On average, patients were 71.42 (±9.97) years old and obese with a BMI of 30.36 (±6.88 kg/m^2^). Twenty-five (62.5%) were operated on the right side. The mean follow-up lasted 2.21 (±0.77) years. Against the Paprosky acetabular bone loss classification system, 1 patient was classified as type I (2.5%), 10 as type IIA (25.0%), 11 as type IIB (35.0%), 2 as type IIC (5%), 11 as type III (35.0%) and 2 as type IIIC (5.0%). Additional demographics are presented in Table 1.

**Table 1:** Demographics

**Age**	71.42 ± 9.97
**Male -*n *(%)**	18 (45.0)
**BMI**	30.36 ± 6.88 kg/m^2^
**Laterality (right) -*n *(%)**	25 (62.5)
**Smoking Status -*n *(%)**	
**Never**	23 (57.5)
**Former**	15 (37.5)
**Current**	2 (5.0)
**Race -*n *(%)**	
**White**	26 (65.0)
**African American**	7 (17.5)
**Other**	7 (17.5)
**ASA Class -*n *(%)**	
**I**	0 (0)
**II**	14 (35.0)
**III**	25 (62.5)
**IV**	1 (2.5)
**Ambulatory status -*n *(%)**	
**No aids**	13 (32.5)
**Cane**	13 (32.5)
**Two crutches**	3 (7.5)
**Walker**	8 (20.0)
**Wheelchair**	3 (7.5)
**Paprosky classification -*n *(%)**	
**I**	1 (2.5)
**IIA**	10 (25.0)
**IIB**	14 (35.0)
**IIC**	2 (5.0)
**IIIB**	11 (27.5)
**IIIC**	2 (5.0)

*ASA* American Society of Anesthesiologists, *BMI* Body mass index, *n* number

### Data search

The electronic medical records in four orthopedic centers were reviewed for demographic data, including age, gender, BMI, ASA scores, smoking status, laterality, indication for surgery, pre- and postoperative ambulatory status and Harris Hip Scores (HHS) scores [10], implants used, surgical complications, hospital length of stay and follow-up period. Additional parameters were documented, including inpatient complications, 90-day postoperative emergency department visits, 90-day postoperative readmissions and all re-revisions data. Acetabular bone loss was categorized according to Paprosky classification [11].

All patients were followed up prospectively for at least 1 year postoperatively. Pre- and postoperative hip stability was evaluated by the performing surgeon. Immediate postoperative anteroposterior and lateral hip radiographs were analyzed along with radiographs taken at three months, twelve months, and annually thereafter. All radiographs were assessed by two orthopedic surgeons who did not perform the surgery. The interface between the porous shell and the host bone was also assessed for initial and progressive radiolucency as well as areas of initial radiolucency that resolved with continued follow-up.

Shell fixation and osteointegration were assessed in relation to De Lee and Charnley zones as described by Moore *et al.* [12]. Three or more of the following signs would indicate radiological osteointegration: superolateral buttress formation, presence of an inferomedial buttress, medial stress shielding, radial trabeculae, and absence of radiolucent lines.

#### Surgical technique

After exposure, in the cases of revision THA, following removal of the failed acetabular component, granulation tissue was removed. The acetabulum was sequentially reamed using dedicated hemispherical reamers. Line-to-line or under reaming by 1 mm was performed based on surgeon's preference and intraoperative bone quality assessment. At this point, the bone stock was reassessed for the possible need of bone grafting to fill the cavitary defects. Trial components were used to assess coverage, impingement, and stability. The fully porous shell was impacted and when satisfactory orientation of the shell was achieved multiple screws were placed. An XLPE liner sized to fit the shell intra-diameter was cemented into the shell. The liner was then pressurized using the appropriately-sized liner impactor head until cement was cured, with any excess cement removed. The femoral stem stability and osteointegration were assessed intraoperatively and the stem was revised at surgeon’s discretion.

**Fig. 2:**
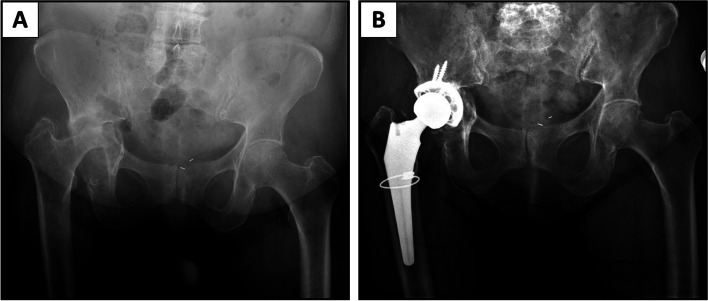
Complex primary THA: **A** Preoperative pelvis AP radiograph of 82 y/o female with previous acetabular fracture and arthrosis. **B** Postoperative AP pelvis radiograph of fully porous cup with cemented XLPE liner and two locking screws

**Fig. 3:**
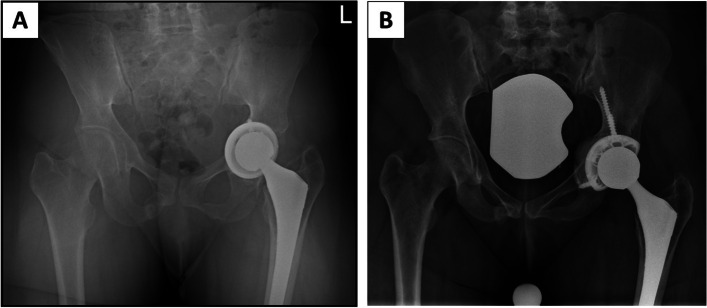
Revision THA: **A** Preoperative pelvis AP radiograph of 72 y/o female with pelvic discontuity and protruded primary acetabular shell. **B** Postoperative AP pelvis radiograph of fully porous cup with cemented XLPE liner and two locking screws

The fully porous revision hemispherical acetabular shell (Smith&Nephew, Memphis, TN, USA), was developed for use in revision cases. The fully porous shell is made from titanium alloy (Ti-6Al-4V) and has been shown to be biocompatible and structurally comparable to cancellous bone. To allow ingrowth, the shell has an inter-connected network of pores with a porosity of up to 80% in the near-surface regions, where the initial fixation will occur and the overall porosity is up to about 67%. Additionally, new variable-angle locking screws can be used to enhance implant stability and minimize micromotion after surgery (Figs. 2 and 3).

### Statistical analysis

Descriptive statistics, including mean, average, range, and standard deviation, were presented for continuous variables. Survivorship was analyzed and presented graphically by using the Kaplan-Meier method. Log rank test was used to calculate *P*-values for the difference between groups. Outcomes and survivorship data were calculated by using time of latest follow-up. All statistical analyses were performed using IBM SPSS software (IBM-SPSS, version 26, Armonk, NY, USA).

## Results

Thirty-four (85.0%) patients underwent rTHA for loosening of an acetabular component of a prior THA (*n* = 22, 55.0%), PJI (*n* = 8, 32.0%), instability (*n* = 2, 5.0%), trunnionosis (*n* = 1, 2.5%) and pseudotumor (*n* = 1, 2.5%). Six patients received cTHA for acetabular medial wall fracture (*n* = 1, 2.5%), femoral neck fracture (*n *= 2, 5.0%), conversion arthroplasty (*n *= 2, 5.0%), and severe destructive OA (*n *= 1, 2.5%) (Table 2).

**Table 2:** Surgery data

**Complex primary THA -*n *(%)**	6 (15.0)
Medial wall fracture	1 (2.5)
Femoral neck fracture	2 (5.0)
Conversion arthroplasty	2 (5.0)
Osteoarthritis	1 (2.5)
**Revision THA**	34 (85.0)
Acetabular aseptic loosening	22 (55.0)
PJI	8 (32.0)
Instability	2 (5.0)
Trunnionosis	1 (2.5)
Pseudotumor	1 (2.5)
**Surgery time (minutes)**	160.8 ± 80.4
**Median fully porous shell - mm**	60 (range, 48–80)
**Median number of screws**	4 (range, 2–8)
**Median femoral head size - mm**	36 (range, 28–36)
**Femoral stem revised**	23 (57.5)
**Bone allograft use**	12 (30.0)
**Intraoperative complications -*n *(%)**	2 (5.0)
Medial femoral wall fracture	1 (2.5)
Calcar fracture	1 (2.5)

*THA* total hip arthroplasty, *PJI* periprosthetic joint infection

The median fully porous shell size was 60 (range, 48–80), number of locking screws 4 (range, 2–8), and femoral head size was 36 (range, 28–36). Twenty-three (57.5%) femoral stems were revised and replaced. Bone allograft was used in 12 (30.0%) cases. Two intraoperative complications were documented as a femur fracture occurred during femoral stem preparation. The average surgery time was 160.8 (±80.4) minutes (Table 2).

One inpatient complication was noted (gastric ulcer that was treated with endoscopy). The mean hospital length of stay was 5.34 (±3.34) days. Three 90-day readmissions were noted: two patients presented to the ED with an acute PJI on POD 15 and 69 respectively. One patient developed a DVT on POD 24 and was treated with anticoagulation therapy (Table 3).

**Table 3:** Patient outcomes

**Inpatient complications -*n *(%)**	1 (2.5)
Gastric ulcer	1 (2.5)
**Hospital LOS (days)**	5.34±3.34
**90-day readmissions –*n *(%)**	2 (7.1)
Acute PJI	2 (5.0)
DVT	1 (2.5)
**Preoperative HHS**	53.87±12.58
**Postoperative HHS**	83.53±12.15
**Discharge Disposition -*n*(%)**	
Home	28 (70.0)
Skilled Nursing Facility	5 (12.5)
Acute Rehab Center	7 (28.0)
**Revision - *n*(%)**	3 (7.5)
Reasons for Revisions	
PJI	2 (7.1)
Aseptic loosening	1 (2.5)
**Osteointegration**	39/40 (97.5)
**Implant migration**	1 (2.5)
**Mean follow up - years**	2.21 (±0.77)

*LOS* hospital length of stay, *PJI* periprosthetic joint infection, *DVT* deep vain thrombosis, *HHS* Harris Hip Score

Harris Hip Scores improved, on average, from 53.87 ± 12.58 preoperatively to 83.53 ± 12.15 postoperatively. One case of aseptic loosening with proximal acetabular shell migration was noted. Two patients received DAIR revision surgery (5.0%) for acute PJI in which the XLPE liner and femoral head were exchanged. One patient underwent revision due to acetabular shell aseptic loosening, the cup was removed and a new shell and liner were implanted with the addition of bone allograft and metal augments (Table 3).

**Fig. 4:**
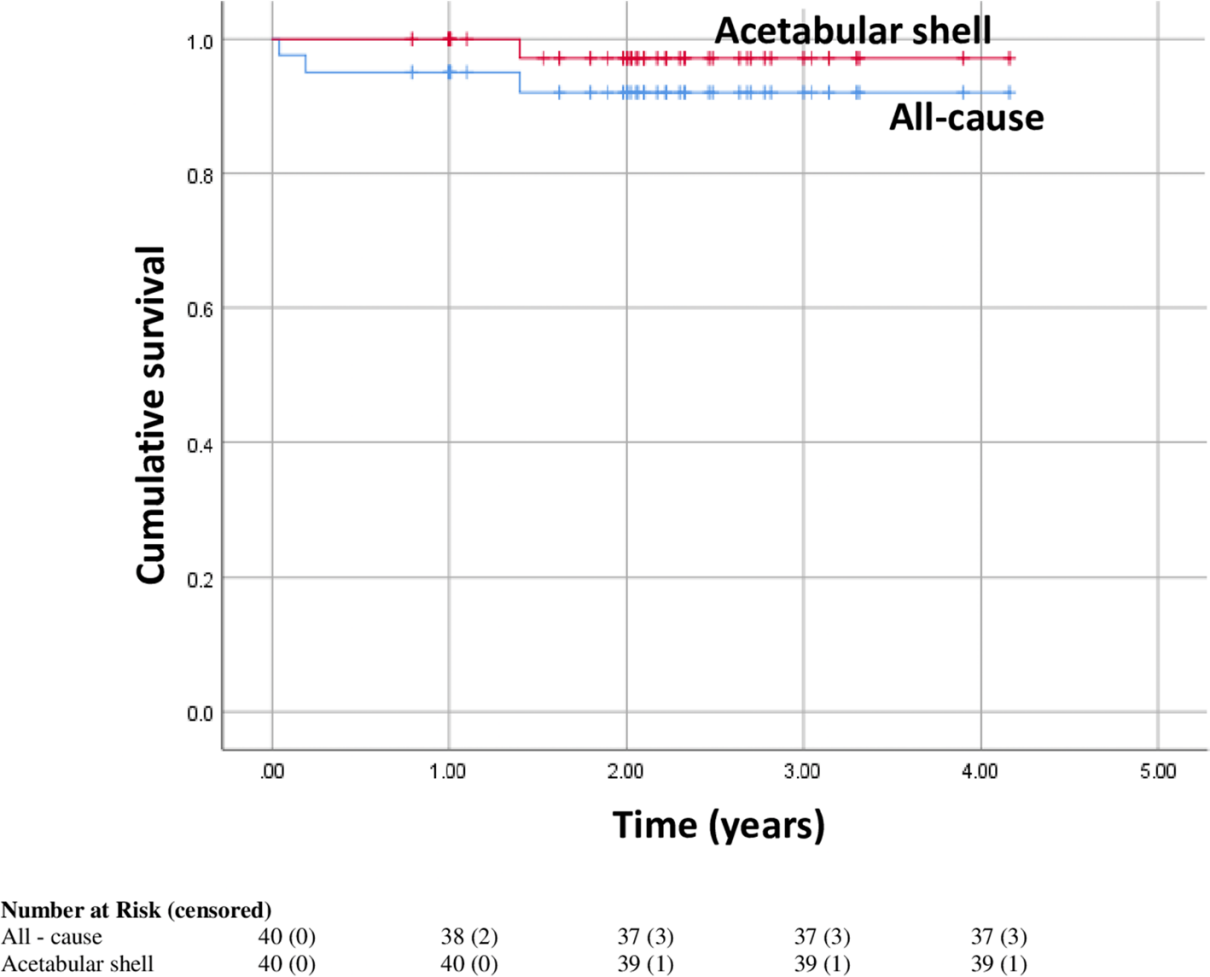
Kaplan-Meier survivorship curve for all cause revision and acetabular cups in revision total hip arthroplasty patients with an at least 1-year follow-up

Kaplan-Meier analysis showed an all-cause revision free survival rate of 95.0% at 6 months and 1 year, and 92.0% at 4 years. Aseptic acetabular shell survival, liner dislocation/loosening, and fracture-free survival was 100% at 6 months and 1 year, and 97.1% at 2 years (Fig. 4).

## Discussion

The main findings of this study were that the combined use of a novel 3D-printed fully porous titanium acetabular shell and cemented XLPE acetabular liner produced excellent freedom from all cause revision and freedom from acetabular revision due to aseptic loosening at short-term follow-up.

The goal of using an uncemented shell in revision THA is to achieve primary mechanical stability in the presence of a favorable biological environment to allow bone ingrowth and oteointegration [13].

Highly porous acetabular shells have bone-like microstructure. Their high coefficient of friction provided a suitable environment for bone ingrowth, bone graft remodeling, and better initial stability [14]. Previous studies reported that titanium acetabular cup designs had a modulus of elasticity between that of cortical and cancellous bone, which transmits physiological load on bone. Thus, this process suppresses implant micromotion, which, in turn, leads to early biological immobilization. All of these contributed to their excellent short to mid-term results that led to the increased use of uncemented shell in revision THA over the last 3 decades [3, 15–18].

The use of highly porous titanium-based acetabular shells in revision total hip surgery has been evaluated in relatively few studies. Delanois *et al*., in a cohort consisting of 35 patients with a minimum of 5-year follow-up, reported that a highly porous titanium acetabular revision shell had excellent all-cause implant survivorship of 91% [19]. Ramappa *et al*. reported excellent short-term results in 43 revision THA cases using a titanium acetabular shell at a mean follow-up of 18.2 months [17]. They had 1 failed shell osteointegration, which was noted at 6 weeks, with 98% of shells being osteointegrated within 3 months after surgery. More recently, Hosny *et al*. examined a cohort of 63 cases of rTHA using a highly porous titanium shell. Their follow-up study (lasting a mean time of 87 months) showed similar excellent results with an implant survivorship of 98.4% for aseptic loosening, only 1 patients requiring re-revision for aseptic loosening [8]. Comparable to these aforementioned studies, our study presented similar rates of freedom of revision of acetabular shell due to aseptic loosening at a minimum follow-up of 1 year. Quinlan *et al*. concluded that aseptic revision surgery following THA and TKA is associated with a significantly increased risk of subsequent PJI within 2 years (4–6 fold) [20]. Similarly, our cohort included 2 patients (7.1%) who underwent a re-revision for prosthetic joint infection.

Greidanus *et al*. concluded that cementless acetabular reconstruction with a hemispherical shell should not be used when biological ingrowth is unlikely to occur, such as in acetabula with severe bone loss. However, the precise amount of host bone loss is controversial [13]. A study by Lackstein *et al*. examined 53 rTHA using trabecular metal shells in the cases with less than 50% bone loss and reported a 96% implant survivorship at a 2-year follow-up [3].

In a cohort involving 138 cementless rTHA shells, Silverton *et al.* reported an excellent radiographic osteointegration rate of 97.1% at a 8-year follow-up [21]. In a more recent cohort of 46 rTHA using uncemented tantalum acetabular shells, Greidnus *et al*. found 100% implant osteointegration at 2-year follow-up as assessed against the Moore criteria [13]. Comparing revision THA patients is complicated due to the differences in the indications for revision, bone defects, and implants used. Nevertheless, recent studies demonstrated similar implant survival rates and freedom from aseptic loosening when comparing trabecular metal to porous titanium cups in rTHA [22, 23]. Similar to those studies, our cohort consisted of patients with moderate to severe bone loss (Paprosky IIA-III), and had an excellent 97.5% osteointegration rate according to the same criteria at 1-year follow up. We believe that this novel construct characteristics and design enable reliable osteointegration despite complex bone defects.

Good results have been reported in the past when cementing a polyethylene liner into a well-fixed acetabular shell in rTHA [24–26]. Cementing a polyethylene liner into a porous acetabular shell enables the surgeon to position the shell at maximal bone and acetabular rim contact, while adjustment to appropriate lateral opening and anteversion is made with the polyethylene liner [13]. Our cohort had 100% freedom from liner loosening or dislocation, showing the safety and efficacy of this surgical technique. Implant survivorship in addition to re-revision-free survivorship are key elements to assess revision THA success. However, it is important to compare clinical and functional outcomes of different highly porous shell designs. In a similar study utilizing 3D-printed fully porous acetabular shell in cTHA, Geng *et al*. demonstrated remarkable HHS improvement from 45.2 ± 4.8 preoperatively to 95.8 ± 6.0 postoperatively [9]. In a cohort containing 35 patients receiving rTHA, Delanois *et al*. reported a mean postoperative Harris Hip Score of 76 points [19]. Similarly, our study results showed improved HHS following surgery, with a mean postoperative score of 85.53 points.

This study is not without limitations. It was of retrospective nature, and as such, possesses certain potential data procurement limitations. In addition, this study had a limited sample size with a short follow-up time. Therefore, we encourage prospective multi-center studies with a larger sample size to be conducted to evaluate the use of highly porous titanium acetabular revision shells in revision THA patients. In addition, this acetabular shell-cemented liner construct should be directly compared to survivorship and clinical outcomes of other widely used implants to assess long-term outcomes. Nevertheless, we believe that the results of this study are encouraging and may be useful for clinical decision-making about the revision THA.

In conclusion, the use of a highly porous titanium acetabular shell with a cemented XLPE liner in complex primary and revision THA demonstrated good early aseptic survivorship, good radiographic outcomes with a low complication rate at a minimum 1-year follow-up. Fully porous shells appear to be an effective choice for patients undergoing cTHA and rTHA with moderate-to-severe bone loss. The short-term results are promising, and we encourage further prospective studies monitoring the long-term outcomes of these implants.

## Data Availability

The datasets used and/or analyzed during the current study are available from the corresponding author on reasonable request.
